# HealthKick: a nutrition and physical activity intervention for primary schools in low-income settings

**DOI:** 10.1186/1471-2458-10-398

**Published:** 2010-07-06

**Authors:** Catherine E Draper, Anniza de Villiers, Estelle V Lambert, Jean Fourie, Jillian Hill, Lucinda Dalais, Zulfa Abrahams, Nelia P Steyn

**Affiliations:** 1 UCT/MRC Research Unit for Exercise Science and Sports Medicine, Sports Science Institute of South Africa, Boundary Road, Newlands, Cape Town, South Africa; 2 Chronic Diseases of Lifestyle Unit, Medical Research Council, Francie van Zijl Drive, Parowvallei, South Africa; 3 Centre for the Social and Environmental Determinants of Nutrition, Knowledge Systems, Human Sciences Research Council, Plein Street, Cape Town, South Africa

## Abstract

**Background:**

The burden of non-communicable diseases, including type 2 diabetes, is growing in South Africa. This country has a complex mix of over- and under-nutrition, especially in low-income communities, and concerning levels of physical inactivity in children and youth. This paper describes HealthKick, a school-based nutrition and physical activity intervention in primary schools in these settings aimed at reducing diabetes risk factors.

**Methods/Design:**

This study includes schools within historically disadvantaged, low-income communities from an urban area close to the city of Cape Town and from two rural areas outside of Cape Town, South Africa. The three Educational Districts involved are Metropole North, Cape Winelands and the Overberg. The study has three phases: intervention mapping and formative assessment, intervention development, and outcome and process evaluation. Sixteen schools were purposively selected to participate in the study and randomly allocated as intervention (eight schools) and control (eight schools).

The primary aims of HealthKick are to promote healthful eating habits and increase regular participation in health-enhancing physical activity in children, parents and teachers, to prevent overweight, and reduce risk of chronic diseases (particularly type 2 diabetes); as well as to promote the development of an environment within the school and community that facilitates the adoption of healthy lifestyles.

The components of HealthKick are: action planning, toolkit (resource guide, a resource box and physical activity resource bin), and an Educators' Manual, which includes a curriculum component.

**Discussion:**

This study continues to highlight the key role that educators play in implementing a school-based intervention, but that developing capacity within school staff and stakeholders is not a simple or easy task. In spite of the challenges experienced thus far, valuable findings are being produced from this study, especially from Phase 1. Materials developed could be disseminated to other schools in low-income settings both within and outside of South Africa. Owing to the novelty of the HealthKick intervention in low-income South African primary schools, the findings of the evaluation phase have the potential to impact on policy and practice within these settings.

## Background

Non-communicable diseases (NCDs), including type 2 diabetes, have become an increasing global concern. The burden of these diseases is growing in South Africa [1] in both urban [2] and rural [3] low-income communities. South Africa has a complex mix of over- and under-nutrition [4], especially in schools situated in low-income communities, and increasing levels of physical inactivity in children [5] and youth [6]. A recent study in a low-income South African setting reported on the co-prevalence of early stunting and adolescent obesity (in girls), and highlighted the concerning nature of these findings in light of obesity and adult short stature as risk factors for type 2 diabetes [7].

Barriers to the promotion of healthy lifestyles in schools in low-income communities include limited resources, the absence of policy relating to healthy lifestyles, and the availability of inexpensive foods of low nutritive value either from tuck shops or street vendors [8].

Physical education was phased out as a stand-alone subject in 2004, and placed in the 'Life Orientation' (LO) learning area as one of its four learning outcomes. Various challenges in implementing physical education within LO have been identified, and the need for capacity development in this area has been emphasised [9].

Substantial literature exists on school-based nutrition and physical activity interventions in a range of settings, and landmark interventions include The Child and Adolescent Trial for Cardiovascular Health (CATCH) [10], Pathways [11] and Action Schools! BC [12]. These interventions have shown positive effects on children's diet and physical activity behaviours [10,13], as well as psychosocial variables, such as self-efficacy, related to nutrition and physical activity [11]. In addition to these effects, these interventions have shown to be feasible, acceptable, and in some cases, sustainable interventions in the school environment [12,14,15].

While these interventions have been implemented in some low-income settings, there is very little literature reporting on school-based interventions in African settings [16-18]. Furthermore, the focus of these studies is the reporting of outcomes and not the description of intervention development and implementation. Such information would facilitate the sharing of lessons learned and could ultimately lead to the implementation of these interventions in other settings. A literature review on school-based interventions provided further focus on specific factors associated with successful interventions. These include a curricular component, physical activity, healthy food-service and family involvement [19].

This paper describes the development of HealthKick, a school-based nutrition and physical activity intervention in primary schools in low-income settings aimed at reducing diabetes risk factors.

## Methods/Design

### Study setting and phases

This study is taking place in the Western Cape province of South Africa. Schools involved are from historically disadvantaged, low-income communities from an urban area close to the city of Cape Town and from two rural areas outside of Cape Town. These schools have all been classified by the Western Cape Education Department (WCED) at the lower end of the poverty index, and fall within three educational districts (one urban, two rural).

This study has three phases, and these will be discussed in the paper:

• Phase 1: Intervention mapping and formative assessment

• Phase 2: Intervention development and implementation

• Phase 3: Outcome and process evaluation

Although implementation forms part of Phase 2, the focus of this paper will be the description of the intervention.

### Intervention aims

The primary aims of the HealthKick intervention are:

• To promote healthful eating habits in children, parents and educators, as a means to reduce the risk of chronic diseases (particularly type 2 diabetes);

• To increase regular participation in health-enhancing physical activity in children, parents and teachers, to prevent overweight, and reduce risk of chronic diseases (particularly type 2 diabetes); and

• To promote the development of an environment within the school and community that facilitates the adoption of healthy lifestyles.

The secondary aims of the intervention are:

• To build capacity of the school staff and stakeholders in the development, implementation and assessment of school-based lifestyle intervention programmes;

• To apply best practice for school-based interventions in a low-resource setting; and

• To present the findings to policy makers together with a plan of wider dissemination of the intervention.

### Theoretical basis of HealthKick

**Figure 1:**
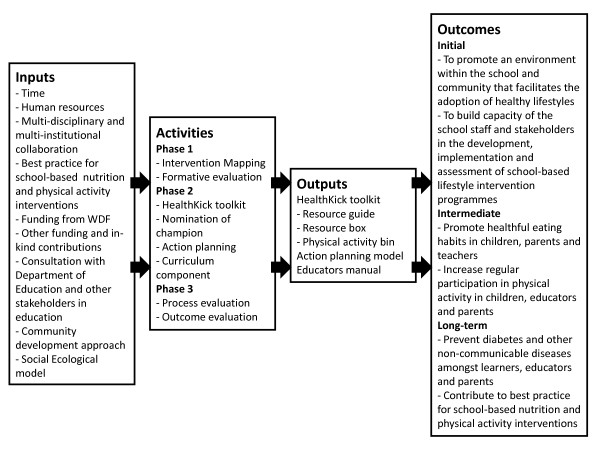
**HealthKick logic model**.

The HealthKick intervention has been developed within the context of the Social Ecological model. In Table 1 the various areas for intervention are linked to the levels of this model. Figure 1 is a logic model of the HealthKick intervention.

**Table 1:** Social Ecological model and HealthKick intervention

Social Ecological levels	Areas for intervention
**Intrapersonal**	Diet, choices and habits, knowledge, self-efficacy and beliefs, fitness levels, awareness
**Interpersonal**	Priorities for parents, encouragement from family and peers, role models
**Organisation (school)**	Resources for physical activity and sport, opportunities for physical activity and sport, encouragement from teachers, implementation of curriculum
**Community**	Socioeconomic circumstances, food insecurity, lack of resources for physical activity and sport, social norms around physical activity and nutrition

### Phase 1: Intervention Mapping and formative assessment

#### Intervention Mapping

Intervention Mapping is a means of systematically applying existing literature, appropriate theories and additional research data in the steps of programme development [20]. In this study, it was used to prioritise key environment and behavioural outcomes regarding nutrition and physical activity, focusing on learners. Table 2 outlines the steps of the Intervention Mapping process that was followed for the HealthKick intervention model. Examples of behavioural outcomes and change objectives are given in Table 3, and Table 4 gives examples of methods and strategies.

**Table 2:** Intervention Mapping for the HealthKick intervention

Intervention Mapping Steps	Programme activities
**1: Needs assessment**	Formative assessment (described below)
	Members of the different planning groups embarked on a series of brainstorming sessions to determine and prioritise the key health problem/s, the behavioural and environmental factors related to the problem/s, and the determinants of these factors
**2: Matrices of change objectives**	Behavioural and environmental outcomes of the intervention were identified
	Performance objectives were specified (required actions for achievement of outcomes)
	Personal and external determinants identified for each performance objective to develop change objectives
	(Change objective = performance objective + determinants)
	Examples of change objectives given in Table 3
**3: Theory-based methods and practical strategies**	These were identified for change objectives; example given in Table 4
**4: Producing programme components and materials**	Programme components and materials described under 'Phase 2' were developed, and include the action planning process and toolkit
**5: Planning programme adoption, implementation and sustainability**	This step is currently underway and consultation with the Western Cape Education Department is key to this part of the process
**6: Planning for evaluation**	This step is currently underway and is described under 'Phase 3'

**Table 3:** Examples of behavioural outcomes and change objectives

Behavioural outcome: Increase learners' participation in physical activity during school time				
	***External determinants***			
**Performance objective:**	**Social support**	**Social norms**	**Access to resources**	**Organisational support**

Be physically active during discretionary time, e.g. break time (recess)	Recognition and reward for those who participate in PA to the best of their ability	Appropriate context - fun, fair, non-judgemental, non-competitive, creativity around competition	Facilities and equipment are available, safe and accessible	Break time is not taken away as punishment
	Encouraged by parents, peers and teachers to be active	Peers are active	Environment conducive to PA during discretionary time	Time for PA is not taken up by other school activities

**Behavioural outcome: Increase the variety of foods eaten daily**				

	***Personal determinants***			
**Performance objective:**	**Knowledge**	**Beliefs**	**Attitudes**	**Self-efficacy & Skills**
Choose to eat a variety of foods according to the SA food-based dietary guidelines (FBDGs)	List the health advantages of eating a variety of food in childhood as well as in adulthood (preventing CDLs)	Believe that obtaining macronutrients from a variety of foods is important for a healthy diet	Enjoy eating a variety of foods daily	Demonstrate the ability to create a daily meal plan that includes all food groups from the FBDGs
	Show awareness of the importance of variety		Value the benefits of eating a variety of foods daily	Express confidence that a variety of food can be eaten every day
	Know the macronutrients and their role			

**Table 4:** Example of methods and strategies

Performance objective	Determinant	Methods	Strategies
**Choose to eat a variety of foods according to the food based dietary guidelines (FBDGs)**	Demonstrate the ability to create a daily meal plan that includes all the food groups according to the FBDGs	• Modeling (Social cognitive theory)	• Teaching through different learning areas in the curriculum
		• Skills training	• Skills practice in role plays and home work assignments
		• Information	• Guiding educators to place the change objective in the curriculum
			• Guiding educators to serve as models for healthy eating practices
**Be physically active during discretionary time, e.g. break time, before school**	Enjoy physical activity and believe that physical activity will make a difference in health and well-being	• Tailoring	Curriculum component that encourages activities such as dance as a means to increase levels of physical activity, and emphasises:
		• Persuasive communication	• How physical activity will make a difference in learners' health and well-being (relating to relevant health issues in their community)
			• That any physical activity is better than nothing
			• That physical activity is important for boys and girls

#### Formative assessment

The formative assessment had the following components:

• Situational analysis of the physical and policy environment relating to nutrition and physical activity in 100 randomly selected schools (50 rural, 50 urban) in the Western Cape. This included a structured interview with the school principal and completion of an observation schedule. The sample size calculation for the situational analysis was based on having a precision of not more than 10% for an estimated percentage of 50%. A sample of 100 schools gave the required precision when estimating the 95% confidence interval for the percentage.

• Health risk assessment of teachers at 83 of the 100 schools (n = 517).

• Health risk assessment of parents at four of the 50 urban schools (n = 50).

• Small-group parent interviews at 22 of the 100 schools (n = 54) [21].

• Questionnaire for Grade 4 learners at four of the 50 urban schools on physical activity and food preferences, perceptions of benefits of physical activity and barriers to school sport, and nutrition knowledge (n = 122).

• Testing of Grade 4 learners (n = 887) from the eight intervention and eight control schools involved a questionnaire on their nutrition and physical activity knowledge, attitudes and behaviour, an individual dietary intake assessment, anthropometric measurements and fitness testing. The questionnaire was developed by the research team, and was informed by questionnaires from Pathways [11] as well as previous local research, validating questionnaires concerning nutrition and physical activity knowledge and self-efficacy in primary school settings [22].

Key findings from the formative assessment are presented in Table 5. Our findings showed that overweight and obesity are not imminent concerns among learners from low-income settings in the Western Cape. However, the formative assessment highlighted that the physical activity and dietary behaviour of these children was not optimal and warranted attention. These findings emphasise the need to intervene with educators not only because of their potential role as agents of change within the school environment, but also because of their generally poor health status. Regarding parents, it is likely that the competing priorities relating to substance abuse, poverty and unemployment have contributed to a culture of their limited involvement within these schools. This was frequently reported by principals during the formative assessment interviews.

**Table 5:** Formative assessment key findings

Top health priorities for learners, as identified by principals (n = 100)	
Unhealthy diet	76%

Lack of physical activity	50%

Underweight	47%

**Top health priorities for parents, as identified by principals (n = 100)**	

Substance abuse	91%

Tobacco use	57%

Unhealthy diet	44%

**Extent to which poverty and unemployment is a concern for learners at your school (n = 100)**	

To a great extent	84%

**Top barriers to adoption of health promotion programmes (n = 100)**	

Lack of financial resources	71%

Inadequate facilities	52%

**Educators' health status (n = 517)**	

Overweight	31%

Obese	47%

Hypertensive	56%

Smoke	80%

High waist circumference	56%

High cholesterol	30%

Low physical activity	77%

#### HealthKick goals

The intervention mapping process (specified behavioural outcomes in particular), including the formative assessment findings, led to the development of the following HealthKick goals for the behaviour of learners, and have guided the intervention thus far. These goals align with the South African food-based dietary guidelines [23].

• Eat a variety of foods every day

• Eat more different kinds of fruit and vegetables every day

• Eat less fat and oily food

• Eat less sugar and sweet foods, such as cakes, doughnuts, sweets, etc.

• Eat a regular healthy breakfast daily

• Bring healthy lunchboxes to school as a daily routine

• Be more physically active during school time

• Be more physically active after school

### Phase 2: Intervention development

Based on the analysis of the formative assessment data, and in consultation with educational authorities, sixteen schools were purposively selected to participate in Phase 2. These schools were randomly placed in intervention and control categories. Owing to the intention to develop capacity within school staff and stakeholders, this intervention has been developed in a way that the team facilitates rather than takes responsibility for the implementation of the intervention. Intervention schools are therefore referred to as 'co-implementation' schools, and control schools as 'self-implementation' schools. Co-implementation schools were asked to nominate a HealthKick 'champion' (an educator or other member of school staff) to act as a focal point for communication with the intervention team. Although the original intention was for this individual to promote healthy lifestyles in their school environment, very few of the champions were able or willing to take on this role. Self-implementation schools have been given access to some printed materials and resources, but the intervention team has not made themselves available to assist with implementing any of the suggestions given.

#### Intervention team and collaborators

The multi-disciplinary intervention team includes individuals from the South African Medical Research Council's Chronic Diseases of Lifestyle Unit, the University of Cape Town/Medical Research Council Research Unit for Exercise Science and Sports Medicine, and the South African Human Sciences Research Council. Areas represented include dietetics, exercise physiology, psychology, epidemiology, biostatistics, qualitative methodology, and physical education. Collaborators include the WCED (at provincial and district levels), the University of the Western Cape's School of Public Health, and the South African Heart and Stroke Foundation. In particular, the cooperation of the WCED district offices (specifically the educational psychologists who represented each of the three education districts) has proven to be invaluable both for the facilitation of the research process and support for the intervention.

#### Action planning

The action planning component of the HealthKick intervention has drawn heavily on Action Schools! BC Planning Guide for Schools and Teachers [24] and the Centres for Disease Control School Health Index: a self-assessment and planning guide [25]. The aim has been to guide the co-implementation school's HealthKick 'champion', principal, staff and individuals affiliated to the school through a process to assess areas for action related to nutrition and physical activity, identify priorities and set feasible goals.

The action planning process was originally designed to cover at least six 'zones':

• Life Orientation (curriculum component)

• Food and nutrition

• Physical activity

• Health promotion for staff

• School policy and environment

• Family and community involvement.

Action planning sessions began in the second half of 2008. The lengthy nature of these sessions and the perception of action planning as additional work by some educators (which emerged in preliminary process evaluation findings) resulted in limited cooperation from schools and educators. This hindered the implementation of the action planning process as it was originally intended. In particular, the HealthKick team found that very few educators were willing to commit to a plan of action, and even fewer attempted to carry out these actions.

**Figure 2:**
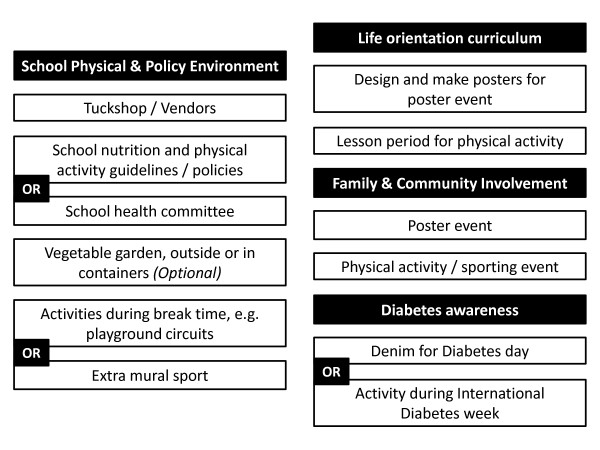
**Action planning zones and areas for action**.

The action planning process was therefore shortened and a more refined model was developed. This had a more focussed format, amalgamating nutrition and physical activity into three of the original 'zones', with the addition of diabetes awareness. Figure 2 outlines these zones as well as the areas for action within these zones. At the start of 2010, schools were required to identify the specific strategies that they would employ to achieve the HealthKick goals within the stipulated areas for action (with some options for certain zones), the individuals within the school who would take responsibility for these strategies, and where possible, a timeframe for completion. Further details are given for the areas for action along with examples of strategies in Table 6.

**Table 6:** Areas for action and examples of strategies

School physical and policy environment	Strategies
Tuckshop/vendors	Enlist the help of the SA Heart and Stroke Foundation, who have developed a tuckshop intervention to assist schools in the promotion of healthier foods
School nutrition and physical activity guidelines/policies	Develop a policy that prohibits the use of physical activity as a form of punishment
	Develop a policy that stipulates the type of food to be sold at fund-raising events
School health committee	Form a representative health committee that helps to address issues relating to nutrition and physical activity (not just general health and safety)
Vegetable garden, outside or in containers	Enlist the help of the SA Department of Agriculture, who have a programme to assist schools in the planting and maintenance of vegetable gardens
Activities during break time, e.g. playground circuits	Use equipment from the physical activity resource bin provided to set up a physical activity circuit around the school grounds
Extra mural sport	Link up with the SA Gymnastics Federation to join their community-based rope skipping programme aimed at primary school children

**Life Orientation curriculum**	

Design and make posters for poster event	Ask learners (Grades 4-6) to make posters that illustrate one or more of the HealthKick goals
Lesson period for physical activity	Ensure that the time allocated to physical activity is 'ring-fenced' for this purpose by drawing on lesson plans and activity suggestions in the resource box provided

**Family and community involvement**	

Poster event	Arrange an event that learners' families and community members can attend to showcase the posters made in Life Orientation
Physical activity or sporting event	Arrange a fun walk in which learners' families and community members can participate to promote the importance of physical activity for health

**Diabetes awareness**	

Denim for Diabetes day	Contact Diabetes SA to arrange this event where learners bring a small cash donation in order to wear denim to raise awareness about diabetes
Activity during International Diabetes week	During International Diabetes week (annually in November) arrange a guest speaker to come and speak on the importance of healthy lifestyles

Health promotion for staff was not included in the refined action planning process, but it was mentioned to the planning teams at each school that this could be one of the focus areas of the health committee. Furthermore, owing to the importance of intervening at this level, a separate intervention for educators is being developed, and will run parallel with the HealthKick intervention.

Instead of going through the action planning process, self-implementation schools received 'HealthKick tips for healthy schools' and the resource guide (explained below).

#### HealthKick toolkit

In order to assist schools with implementing strategies selected as part of the action planning process, co-implementation schools were given a HealthKick toolkit. The toolkit comprises a resource guide, a resource box and a physical activity resource bin. The resource guide is a printed manual containing information about existing resources from government, non-governmental organisations and industry about nutrition, physical activity and diabetes. The physical activity resource bin contains basic equipment such as skipping ropes, balls, bean bags, stopwatches and whistles. The resource box contains 6 magazine holders in a stow-away file box with the following contents, all of which are available locally or via internet access:

• Nutrition lesson plans

• Nutrition information

• Physical activity lesson plans and activities

• Physical activity information

• Information on chronic diseases

• School policy and health environment

#### Educators' Manual

The HealthKick Educators' Manual was developed as an essential component of the intervention. It aims to collate and present developed materials into a tool that can be distributed to schools. It constitutes the curriculum component of the HealthKick intervention and also contains information pertaining to the action planning process, the resource guide and various other resources, such as the South African food-based dietary guidelines.

Through Intervention Mapping, it became evident that to ensure sustainability, HealthKick would need to align with Life Orientation (LO). This was supported by formative assessment findings that identified educators' capacity as a barrier to the implementation of school-based health promotion programmes, as well as other research in this area [9].

Within South Africa's National Curriculum, LO has the following learning outcomes: health promotion, social development, personal development and physical development and movement (see Additional File 1). HealthKick goals for learners (mentioned earlier), in collaboration with a LO curriculum expert, were linked with assessment standards for these learning outcomes for Grades 4 - 6, such as 'Investigates menus from various cultures and suggests plans for healthy meals'. These were divided into knowledge, skills and values required to fulfil the assessment standards. Furthermore, activities (classroom and home-based) and assessments were suggested. During the course of 2009, workshops were held with educators from co-implementation schools to explain the relevance of HealthKick to LO, and indicate ways in which HealthKick supports its implementation.

In 2010, the curriculum documents compiled were consolidated and included in the HealthKick Educators' Manual. This manual is a vital outcome of this intervention as it has the potential to be disseminated by the WCED owing to its alignment with the LO curriculum.

### Phase 3: Outcome and process evaluation

#### Outcome evaluation

Testing conducted with Grade 4 learners for the formative assessment was repeated to establish baseline data for the outcome evaluation. This testing will be repeated at 18 (Grade 5) and 24 months (Grade 6) on the same cohort. Both multi-level (repeated measures) and cohort analysis will be conducted with the Grade 4, 5 and 6 questionnaire data.

The sample size for the formative assessment and baseline testing of learners was based on the changes the HealthKick team hoped to be able to detect in response to the intervention. For example, in order to detect changes in leisure time moderate-to-vigorous physical activity (MVPA) similar to those demonstrated in other school-based interventions, we would require about 90 learners for the intervention and control groups, respectively [26]. Similarly, to be able to detect a significant improvement in nutrition and physical activity knowledge (~8%), at least 106 learners per group would be required [27]. All calculations were done for 80% statistical power at an alpha level of 0.05, suggesting that the study is sufficiently powered for detecting increased health knowledge, and increased minutes of leisure time MVPA.

#### Process evaluation

Process evaluation will include the evaluation of the feasibility and acceptability of the action planning process and the extent of use of the toolkit (resource guide, resource box, physical activity resource bin) at these schools. At self-implementation schools, similar methods will be used to analyse the extent to which they used the resource guide and 'HealthKick tips for healthy schools'. In terms of the strategies selected by co-implementation schools, the reach, implementation fidelity, perceived success of strategy, and factors contributing to and/or hindering success of these strategies will also be assessed. A mixed methods approach will be used to conduct the process evaluation.

Ethical approval for this study was obtained from the Research Ethics Committee in the Faculty of Health Sciences, University of Cape Town (REC REF: 486/2005). In addition, approval for intervention in primary schools was obtained from the Western Cape Education Department. Written consent was obtained from all research participants (formative assessment, and process and outcome evaluations). Parental consent was obtained for learners participating in the research.

## Discussion

This paper describes the three phases of the HealthKick study that aims to develop, implement and evaluate a nutrition and physical activity intervention in low-income primary schools aimed at reducing risk factors for the development of type 2 diabetes. The development of the HealthKick intervention was based on the Intervention Mapping process [20], and a formative assessment phase. This development process was largely iterative as the intervention has been adapted and refined as a result of interaction with schools throughout its implementation. It is likely that this continuous improvement approach will persist in the evaluation phase of the study.

### Lessons learned

The Intervention Mapping approach provided a useful foundation for the development of the HealthKick intervention, particularly the development of the HealthKick goals. Furthermore this approach has contributed towards the integrity of the study and instruments that have been developed. However, we believe that the efficacy of the approach may require further interrogation in light of the time and effort taken to complete the process and the complexity it entailed. This experience is similar to others who have used components of the Intervention Mapping protocol in the development of school-based interventions [28-30].

This study continues to highlight the key role that educators' play in implementing a school-based intervention, but that developing capacity within school staff and stakeholders is not a simple or easy task. Throughout the development and implementation of the HealthKick intervention, the team has been constantly aware of the need to balance educators' sense of autonomy with the need to be prescriptive about what needs to get done. This has been particularly relevant during the action planning process and to some extent the nomination of champions.

While the team initially erred on a less prescriptive approach, giving educators a wide range of options, the need to see something implemented in the school environment has led to fewer options and a more prescriptive approach. Preliminary process evaluation results suggest that the original action planning process has therefore proved not to be feasible in co-implementation schools, although the more recent response from schools (in 2010) has been more encouraging and our perseverance with schools is beginning to bear fruit. However, the outcome of this process in terms of strategies is yet to be measured and the extent to which action planning will lead to changes within the school environment remains to be demonstrated.

Parents have played a very small role in the HealthKick intervention thus far, although this should change as a result of strategies to encourage family and community involvement such as poster events and fun walks. We will need to continue to take cognisance of the influence of the home environment on healthy eating and physical activity, bearing in mind the challenges of substance abuse, poverty and unemployment and the various health consequences and correlates of these issues. Plans are on the way to continue the work done with parents [21] and develop a cost-effective social marketing intervention to educate family and community members about the influences of media and marketing on children's food choices, and to use health communication to change the home environment as it relates to nutrition and physical activity.

In conclusion, in spite of the challenges experienced thus far, valuable findings have been produced from this study, especially from Phase 1. Materials developed could be disseminated to other schools in low-income settings both within and outside of South Africa. Owing to the novelty of the HealthKick intervention in low-income South African primary schools, the findings of the evaluation phase have the potential to impact on policy and practice within these settings.

## Competing interests

The authors declare that they have no competing interests.

## Authors' contributions

NPS and EVL (principal investigators) conceptualised the study. AdV is the project manager of the study. CED assists with the evaluation component of the study. AdV, CED, JH, JF, LD and ZA were involved in data collection and implementation of the intervention. All authors participated in the IM process and contributed to the development of the intervention. All authors participated in the writing of the paper, provided comments on drafts and approved the final version.

## Pre-publication history

The pre-publication history for this paper can be accessed here:

http://www.biomedcentral.com/1471-2458/10/398/prepub

## Supplementary Material

Additional file 1 **Life Orientation learning outcomes and assessment standards for Grades 4 - 6**. Click here for file
