# Supplementary material for: HealthKick: a nutrition and physical activity intervention for primary schools in low-income settings
Source: BMC Public Health. 2010 Jul 6;10:398. doi: 10.1186/1471-2458-10-398 (PMC2910683; doi:10.1186/1471-2458-10-398)
Supplement: Additional file 1 — Life Orientation learning outcomes and assessment standards for Grades 4 - 6. [file 1471-2458-10-398-S1.DOC]

| **Learning Outcome 1: HEALTH PROMOTION**  **The learner will be able to make informed decisions regarding personal, community and environmental health.** | | |
| --- | --- | --- |
| **Grade 4** | **Grade 5** | **Grade 6** |
| **Assessment Standards**  **We know this when the learner:** | **Assessment Standards**  **We know this when the learner:** | **Assessment Standards**  **We know this when the learner:** |
| **4.1.1  Investigates menus from various cultures and suggests plans for healthy meals.** | 5.1.1  Explores and reports on ways to protect the quality of food and water in various contexts. | 6.1.1 Interprets food labels and critically discusses health effects of listed ingredients. |
| **4.1.2  Explores and reports on links between a healthy environment and personal health.** | 5.1.2  Investigates a local environmental health problem using different data sources, and plans a strategy to address the problem. | 6.1.2  Participates in a problem-solving activity to address an environmental health issue to formulate environmentally sound choices and/or actions. |
| **4.1.3  Explains children’s health rights and responsibilities, and suggests ways in which to apply these in a familiar situation.** |  |  |
| **4.1.4  Lists and explains traffic rules relevant to road users.** |  |  |
|  | 5.1.5  Recognises the symptoms and causes of locally occurring diseases and discusses prevention strategies. | 6.1.5  Explains causes of communicable diseases (including HIV/AIDS) and available cures, and evaluates prevention strategies, in relation to community norms and personal values. |
|  | 5.1.6  Explains the individual health and social effects of substance abuse. | 6.1.6  Identifies different forms of abuse and suggests strategies to deal with them. |

| **Learning Outcome 2: SOCIAL DEVELOPMENT**  **The learner will be able to demonstrate an understanding of and commitment to constitutional rights and responsibilities, and to show an understanding of diverse cultures and religions.** | | |
| --- | --- | --- |
| **Grade 4** | **Grade 5** | **Grade 6** |
| **Assessment Standards**  **We know this when the learner:** | **Assessment Standards**  **We know this when the learner:** | **Assessment Standards**  **We know this when the learner:** |
| **4.2.1  Discusses children’s rights and responsibilities as stipulated in the South African Constitution.** | 5.2.1  Applies children’s rights and responsibilities to a range of problem situations. | 6.2.1  Reflects on own application of children’s rights as stated in the South African Constitution. |
| **4.2.2  Identifies and explains stereotype, discrimination and Bias.** | 5.2.2  Discusses instances of stereotype, discrimination and bias, and presents a plan to deal with them in own local context. | 6.2.2 Discusses effects of gender stereotyping, sexism and abuse on personal and social relationships. |
| **4.2.3  Compares the relationship between elders and children in a variety of situations in different cultural contexts.** |  |  |
| **4.2.4  Comments on moral lessons selected from the narratives of a range of cultural groups in South Africa.** | 5.2.4  Discusses the contributions of women and men in a range of cultural contexts. | 6.2.4  Interprets the meaning and personal and social significance of important stages in the individual’s life in a variety of cultures. |
| **4.2.5  Discusses significant places and buildings in a variety of religions.** | 5.2.5  Discusses festivals and customs from a variety of religions in South Africa. | 6.2.5  Discusses the dignity of the person in a variety of religions in South Africa. |
|  | 5.2.6  Discusses the significance of friends in times of tragedy and change. |  |
|  |  | 6.2.7  Discusses and evaluates the significance of a nation-building programme associated with a national day. |

| **Learning Outcome 3: PERSONAL DEVELOPMENT**  **The learner will be able to use acquired life skills to achieve and extend personal potential to respond effectively to challenges in his or her world.** | | |
| --- | --- | --- |
| **Grade 4** | **Grade 5** | **Grade 6** |
| **Assessment Standards**  **We know this when the learner:** | **Assessment Standards**  **We know this when the learner:** | **Assessment Standards**  **We know this when the learner:** |
| **4.3.1 Identifies own strengths and those of others, and explains how to convert less successful experiences into positive learning experiences.** | 5.3.1 Identifies personal successes and develops an action plan for continued positive self-concept formation. | 6.3.1 Reflects on own abilities, aptitudes, interests and strengths as well as body image. |
| **4.3.2 Explains why other persons’ bodies should be respected.** | 5.3.2 Shows an understanding of and respect for body changes. |  |
| **4.3.3 Considers and interprets the emotions of others.** | 5.3.3 Appropriately expresses and copes with a range of emotions. |  |
| **4.3.4 Demonstrates the ability to select and apply useful responses in conflict situations.** | 5.3.4 Explores and evaluates ways of responding effectively to violent situations and contexts. | 6.3.4 Demonstrates peacekeeping and mediation skills in different conflict situations. |
| **4.3.5 Reflects on and learns from own personal experience of working in a group.** | 5.3.5 Reflects on how feedback can be given and received. | 6.3.5 Explains what has been learned by reflecting on an experience related to self-management skills. |
| **4.3.6 Applies appropriate study skills.** | 5.3.6 Develops and implements a personalised study method. |  |
|  |  | 6.3.7 Explains how to respond to peer pressure in different situations. |
|  |  | 6.3.8 Describes and selects a range of problem-solving skills for different contexts. |

| **Learning Outcome 4: PHYSICAL DEVELOPMENT AND MOVEMENT**  **The learner will be able to demonstrate an understanding of, and participate in, activities that promote movement and physical development.** | | |
| --- | --- | --- |
| **Grade 4** | **Grade 5** | **Grade 6** |
| **Assessment Standards**  **We know this when the learner:** | **Assessment Standards**  **We know this when the learner:** | **Assessment Standards**  **We know this when the learner:** |
| **4.4.1 Participates in a variety of simplified invasion games.** | 5.4.1 Explores a range of target games. | 6.4.1  Applies relevant concepts in a variety of striking and fielding games. |
| **4.4.2  Demonstrates different ways to locomote, rotate, elevate and balance, using various parts of the body, with control.** | 5.4.2 Performs movement sequences that require consistency and control in smooth and continuous combinations. | 6.4.2  Demonstrates refined sequences emphasizing changes of shape, speed and direction through gymnastic actions. |
| **4.4.3  Demonstrates basic field and track athletics techniques.** | 5.4.3  Demonstrates a range of field and track athletics techniques. |  |
| **4.4.4  Performs rhythmic movements with awareness of posture.** | 5.4.4  Performs rhythmic movements and steps with attention to posture and style. | 6.4.4  Performs rhythmic patterns of movement with co-ordination and control. |
| **4.4.5  Identifies dangers and responsible safety measures in and around water.** | 5.4.5 Demonstrates knowledge of safety measures in and around water. |  |
|  |  | 6.4.6  Applies basic First Aid in different situations. |
|  |  | 6.4.7  Participates in a physical fitness programme designed to develop particular aspects of fitness. |
